# A Novel Biodegradable and Thermosensitive Poly(Ester-Amide) Hydrogel for Cartilage Tissue Engineering

**DOI:** 10.1155/2018/2710892

**Published:** 2018-12-19

**Authors:** Tsai-Sheng Fu, Yu-Hong Wei, Po-Yuan Cheng, I-Ming Chu, Wei-Chuan Chen

**Affiliations:** ^1^Department of Orthopaedic Surgery, Chang Gung Memorial Hospital in Keelung, Chang Gung University, School of Medicine, Taoyuan, Taiwan; ^2^Graduate School of Biotechnology and Bioengineering, Yuan Ze University, Chung-Li, Taoyuan 320, Taiwan; ^3^Department of Chemical Engineering, National Tsing Hua University, Hsinchu, Taiwan

## Abstract

Thermosensitive hydrogels are attractive alternative scaffolding materials for minimally invasive surgery through a simple injection and in situ gelling. In this study, a novel poly(ester-amide) polymer, methoxy poly(ethylene glycol)-poly(pyrrolidone-co-lactide) (mPDLA, P3L7) diblock copolymer, was synthesized and characterized for cartilage tissue engineering. A series of amphiphilic diblock copolymers was synthesized by ring-opening polymerization of mPEG 550, D,L-lactide, and 2-pyrrolidone. By dynamic light scattering analysis and tube-flipped-upside-down method, viscoelastic properties of the mPDLA diblock copolymer solution exhibited sol-gel transition behavior as a function of temperature. An in vitro degradation assay showed that degradation acidity was effectively reduced by introducing the 2-pyrrolidone monomer into the polyester hydrogel. Besides, mPDLA exhibited great biocompatibility in vitro for cell encapsulation due to a high swelling ratio. Moreover, cell viability and biochemical analysis proved that the mPDLA hydrogel presented a great chondrogenic response. Taken together, these results demonstrate that mPDLA hydrogels are promising injectable scaffolds potentially applicable to cartilage tissue engineering.

## 1. Introduction

Osteoarthritis (OA) is statistically the most common degenerative joint disease in elderly people [1]. To diagnose and treat OA is a critical issue, as three quarters of elderly people are suffering from various degrees of OA [2]. To repair defects in the articular cartilage is difficult because of their avascular nature, easy formation of fibrocartilage, and limited number of chondrocytes to facilitate the recovery [3]. Regenerative medicine is being increasingly developed in recent years. Autologous chondrocyte implantation represents one of the first tissue engineering applications for the regeneration of articular cartilage surface [4]. Tissue engineering approach using appropriate scaffolds might be able to induce a correct type of cartilage from different cell sources such as bone marrow-derived stem cells [5]. Three dimensional scaffolds can be used to expand the number of chondrocytes without dedifferentiation. An ideal scaffold for cartilage tissue engineering should be biocompatible and biodegradable and should have adequate degradation and adsorption rates for tissue replacement [5].

Recently, the concept of injectable scaffolds was translated into clinic, and injectable scaffolds were applied easily to patients' affected region, to reduce pain [6]. This approach is performed through minimally invasive surgery. Among injectable scaffolds, the cross-linking of the thermosensitive hydrogel is a physical reaction that forms the gel structure by intramolecular bonding. Besides, physical cross-linking reaction does not require the addition of a cross-linking reagent, which might cause toxicity [6]. The properties of thermosensitive hydrogels exhibit a suitable sol-gel transition behavior, namely the polymer is in a solution state below room temperature while gelling at body temperature [6]. Hence, to apply thermosensitive hydrogels in tissue engineering, the property of in situ gel-forming behavior is crucial.

One of the most common thermosensitive hydrogels is the polyester polymer PEG-PLGA [7, 8]. Our previous studies revealed that the triblock copolymer PEG-PLGA-PEG, with molecular weights of 550 and 2805 for PEG and PLGA, respectively, showed great thermosensitive properties [9]. To synthesize the triblock copolymer, it required the addition of the cross-linking reagent diisocyanate. Nevertheless, the residual diisocyanate after polymer synthesis might cause cell toxicity. To solve this problem, the mPEG-PLGA diblock copolymer with molecular weights (MW) of 550 and 1405 was used in an earlier study and showed great thermosensitive properties. However, the acidity from PLGA degradation in tissue might result in an inflammatory response.

This study tried to synthesize a novel diblock copolymer by replacing glycolic acid of the mPEG-PLGA diblock copolymer with 2-pyrrolidone to reduce acidity and increase hydrophilicity. Based on the above reasons, a series of amphiphilic poly(ester-amide) diblock copolymers with thermosensitive properties was synthesized by ring-opening polymerization of mPEG 550, D,L-lactide (LA), and 2-pyrrolidone (PD). Under a fixed ratio of PD and LA (PD/LA = 30/70), mPEG-poly(pyrrolidone-co-lactide) (mPDLA) with hydrophobic segments (MW 1105, 1405, and 1705) was synthesized for assessment of the optimal property of the thermosensitive hydrogel. In addition, physical properties and biocompatibility were also examined for the potential application of the thermosensitive hydrogel in cartilage tissue engineering.

## 2. Materials and Methods

### 2.1. Synthesis of mPEG-Poly(Pyrrolidone-Co-Lactide) Diblock Copolymers

**Figure 1:**
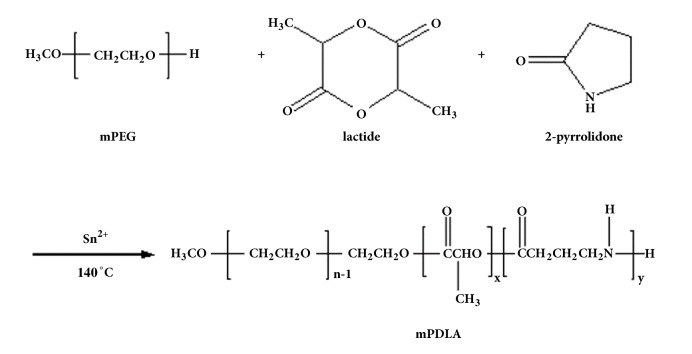
The schematic drawing for the polymerization process of these three monomers.

All materials were purchased from Sigma-Aldrich (Sigma, St. Louis, MO, USA) and used as received unless otherwise specified. mPEG-poly(pyrrolidone-co-lactide) (mPDLA) with hydrophobic segments (MW 1105, 1405, and 1705) was synthesized by ring-opening polymerization of D,L-lactide and 2-pyrrolidone using mPEG 550 as the initiator and stannous 2-ethyl hexanoate as the catalyst. All glassware used was dried under vacuum at 40°C, and polymerization was carried out for 8 h at 140°C. After copolymerization, the crude product was dissolved with three times volume of dimethyl sulfoxide (DMSO) and dialyzed at 4°C for 4 days to remove the unreacted monomer. The final product was dried under vacuum and stored at -20°C. The schematic drawing for the polymerization process of these three monomers was shown on Figure 1.

### 2.2. Characterization of mPEG-Poly(Pyrrolidone-Co-Lactide) Diblock Copolymer by NMR, FTIR, and GPC

The molar ratio of mPEG to polyester was analyzed by a 500-MHz NMR spectrometer (Bruker, Bremen, Germany) at room temperature with CDCl_3_ as the solvent and tetramethylsilane as the internal standard. NMR spectroscopy was performed using ^1^H-NMR was employed to calculate molecular weight and structure. FTIR measurements were obtained using the Perkin-Elmer system 2000 (Bruker) with KBr pellets. The transmittance data were collected for analysis. Molecular weights and their distributions were determined by GPC (Jasco, Tokyo, Japan), with tetrahydrofuran used as the solvent with a flow rate of 1.0 mL/min.

### 2.3. Analysis of Dynamic Light Scattering (DLS)

The DLS was performed on samples of polymer micelles to determine the size of the structures formed in phosphate buffered saline (PBS, Sigma) at a concentration of 1 mg/mL. Three measurements were performed, each consisting of 10 runs for 10 seconds. All experiments were performed at 4°C and 25°C and equilibrated for 3 minutes. Prior to analysis, samples were passed through a 0.45 *μ*m filter.

### 2.4. Swelling Ratio Test

The swelling of mPDLA diblock copolymer hydrogels was measured in PBS at 37°C. mPDLA diblock copolymer solution (20 wt %) in PBS was placed in a 37°C incubator to allow gelation. The hydrogel was immersed in PBS and kept at 37°C for 2 h, 4 h, and 8 h until equilibrium swelling had been reached. The swollen hydrogels were removed from the incubation medium, wiped with tissue paper to remove the surface water at 37°C, and weighed immediately (Ws). Dry hydrogels were weighed (Wd) after being quickly frozen at −80°C and lyophilized. The swelling ratio (SR) was calculated using the following:

(1) $$\begin{array}{c} SR=\frac{\left(Ws-Wd\right)}{Wd}. \end{array}$$

in which Ws is the weight of hydrogel after swelling and Wd is the weight of dried hydrogel after swelling.

### 2.5. Thermosensitive Hydrogel Sol-Gel Transition

The sol-gel transition profiles were performed according to Lai et al., 2014 [10]. Briefly, the sol-gel transition profiles were investigated using the ‘tube-flipped-upside-down' method. All samples were prepared in Eppendorf tubes and incubated at 4°C until the temperature achieved equilibrium. Different weight concentrations of the mPDLA diblock copolymer solution (5 wt%, 10 wt%, 15 wt%, 20 wt%, 25 wt%, and 30 wt%) were prepared and incubated at 4°C. The temperature was then raised by intervals of 2°C and maintained for 5 min before each sampling. The Eppendorf tubes were flipped upside down for 30 s to observe any movement to determine the sol-gel status. The temperatures of sol-to-gel and gel-to-sol transformation were recorded to produce a phase diagram using Gaussian regression.

### 2.6. Mechanical Properties of Sol-Gel Transition

The mechanical properties of hydrogels were performed according to Lai et al., 2014 [10]. Briefly, the mechanical properties of hydrogels were measured using a rheometer (TA Instruments, DE, USA) with the temperature controller set from 5°C to 40°C at a heating rate of 2.2°C/min. A polymeric solution of 15 and 20 wt% were added to the instrument to analyze the rheological behavior of the sol-gel transition. The temperature of initial G' (storage modulus) higher than G” (loss modulus) was defined as the phase transition temperature. The viscosity was measured along with the heating process. The experiments were done in triplicate.

### 2.7. *In Vitro *Degradation of the Hydrogel

The* in vitro* degradation behavior of the hydrogels was performed according to Lai et al., 2014 [10]. Briefly, the mPDLA diblock copolymer solution (0.3 mL of 20 wt% concentration) was injected into release bottles and incubated in a shaking bath at 37°C. After 30 min, 0.7 mL of PBS solution (pH 7.4) was added to the formed gels. At a predetermined time (0, 1, 3, 5, 7, 9, 13, 16, 20, 25, and 31 days), three samples were taken out to assess weight loss. The remaining gels were freeze-dried until a constant weight was achieved. The percentage of residual weight was calculated as follows:

(2) $$\begin{array}{c} \left(\;\frac{Wd}{Wo}\;\right)\times 100\%. \end{array}$$

in which Wd is the residual weight at the predetermined time and Wo is the original weight of the dried composite gel. The pH value of the solution containing degraded byproducts was measured using a pH meter (Shindengen, USA) over a 31-day period.

### 2.8. Isolation and Culturing of Chondrocytes

Chondrocytes were isolated from knees of New Zealand rabbits. The cartilage tissue was minced with scissors, cut into 1 mm3 fragments, and washed thoroughly with PBS. One milliliter of collagenase solution (4 mg/mL) was added to each fragment and digestion was carried out for 5 h at 37°C. Digested tissue was passed through a 100-*μ*m filter and centrifuged to obtain chondrocyte pellets. Chondrocytes were washed with PBS, counted, and cultured in chondrogenic medium (Dulbecco's Modified Eagle Medium (DMEM, Thermo Fisher Scientific, MA, USA) with Insulin-Transferrin-Selenium+1 (ITS+1) Premix (1%; Becton Dickinson, NJ, USA), bovine serum albumin (BSA; 1.25 mg/mL; Sigma), pyruvate (1 mM; Sigma), ascorbate 2-phosphate (0.15 mM; Sigma), dexamethasone (10^–7^ M; Sigma), and TGF-*β*3 (10 ng/mL; R&D Systems, MN, USA) at 37°C with 5% CO_2_.

### 2.9. Cell Encapsulation

Chondrocytes were harvested by trypsin, resuspended in DMEM (Thermo Fisher Scientific), and centrifuged to form pellets (1 x 10^6^ cells per pellet). A total of 200 *μ*L of 30% (w/v) copolymer solutions was mixed homogenously with resuspended cells and transferred to a 48-well plate to form solid hydrogels. One milliliter of pre-warmed medium was added after 5 min, and samples were incubated. Medium was changed every 2 days.

### 2.10. Cell Viability Measurement

Viability of chondrocytes encapsulated in hydrogels was studied using the thiazolyl blue tetrazolium bromide (MTT; Sigma) method. MTT reagent (200 *μ*L, 2.5 mgmL) was added to cell-laden hydrogels and incubated for 3 h. The supernatant was aspirated, and 200 *μ*L of DMSO was added to dissolve the purple violet crystals. Samples were read after 30 min of incubation at 490 nm. Visualization of cells was performed using a LIVE/DEAD staining kit. At a predetermined time (2 and 4 weeks), the cell culture medium was then withdrawn and mixed with fluorescent dye (4 *μ*L of calcein-AM, 2 *μ*L of ethidium homodimer, and 1 mL of sterile PBS) for 30 min in the dark. Inverted fluorescence microscopy (Zeiss, Göttingen, Germany) was used to determine cell viability, with green-stained cells being living cells and red-stained cells being dead cells. mPEG-PLGA hydrogel was used as a control and compared with the mPDLA hydrogels.

### 2.11. Biochemical Assay

The biochemical assay was performed according to our previous article [5, 11]. Briefly, DNA count and glycosaminoglycans (GAGs) quantification in the hydrogels were measured. Briefly, cell-laden hydrogels were lyophilized prior to the assay. Dried hydrogels were digested with papain solution (Sigma) at 65°C for 16 h. The DNA content in the scaffolds was determined using PicoGreen dsDNA Quantification Kit (Molecular Probes, OR, USA) and fluorometric quantification, according to the manufacturer's instructions. The GAG content in the scaffold was assayed using the 1,9-dimethyl methylene blue solution (DMMB) method. The optical density was determined on an ELISA reader at 525 nm (Bio-Tek Instruments, VT, USA). The measured values were corrected by values obtained from the same scaffold without cells cultured for the same period.

### 2.12. Statistical Analysis

The statistical analysis was performed according to our previous article [5]. Briefly, each experiment was replicated three times and values were expressed as mean ± standard deviation (SD). Statistical analysis was done by analysis of variance (ANOVA) to determine statistical significance. Statistical significance was assumed at p < 0.05. For the mechanical properties of sol-gel transition, the maximal values were analyzed using two-tailed Student's t test, and p < 0.05 was designated as statistically significant.

## 3. Results

### 3.1. Synthesis and Characterization of mPDLA Copolymers

**Figure 2:**
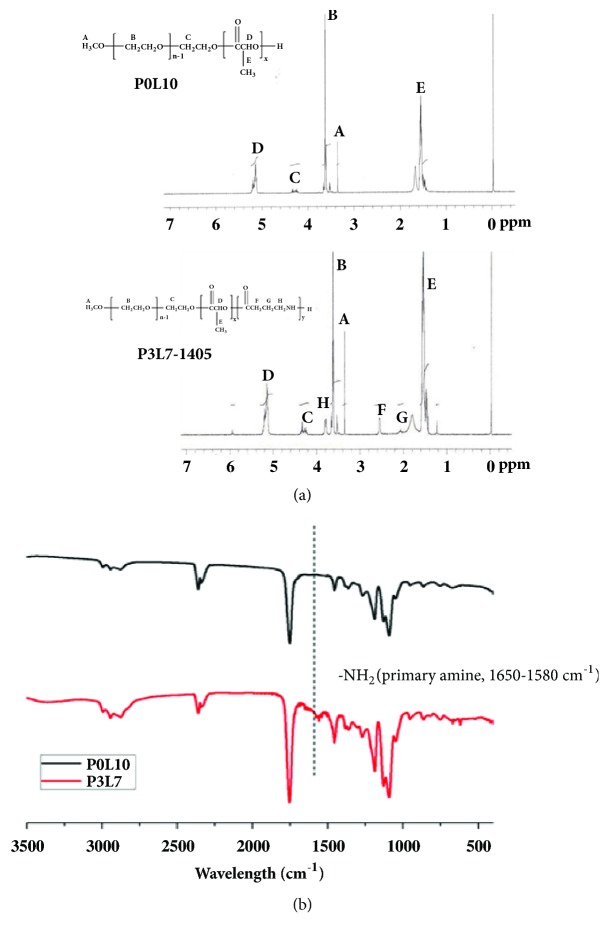
Spectrums of (a) ^1^H-NMR and (b) FTIR on P3L7-1405 and P0L10.

mPEG-poly(ester-amide) copolymers with great injectable and thermosensitive characteristics were synthesized by ring-opening polymerization with the terminal alcohol of mPEG as the initiator. The ratio of D,L-lactic acid (LA) and 2-pyrrolidone (PD) was kept constant at 70:30. The molar ratio of mPEG to polyester was calculated from the ^1^H-NMR (Figure 2(a)) peak area ratio of mPEG methylene protons to characteristic signals of protons on D,L-lactic acid and 2-pyrrolidone. However, the practical reaction ratio of D,L-lactic acid and 2-pyrrolidone was lower than the expected reaction ratio, according to the ^1^H-NMR results in Table 1. Figure 2(a) displayed three differential proton peaks (F, G, and H) in between P0L10 and P3L7 (i.e., [PD]/[LA]=0/100) and P3L7 ([PD]/[LA]=30/70) indicated that those protons were contributed from 2-pyrrolidone. Synthesized copolymers exhibited molecular weights were like the theoretical value with relatively low polydispersities (Table 1) as determined by gel permeation chromatography (GPC). The spectral result of Fourier transform infrared spectroscopy (FTIR, Figure 2(b)) further showed a differential peak at wavenumber 1650 cm^−1^ when comparing P0L10, which was indicated a primarily amine group. This primary amine was contributed from the amide carbonyl structure in PD monomers. According to spectrums of ^1^H-NMR and FTIR, the mPDLA copolymers showed successful synthesized among the monomers of D,L-lactic acid and 2-pyrrolidone and mPEG.

**Figure 3:**
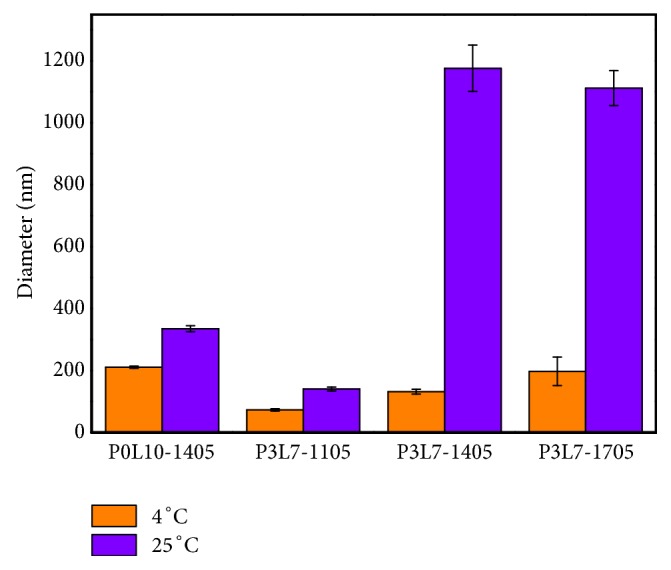
The size of micelle aggregation of P0L10, P3L7-1105, P3L7-1405 and P3L7-1705 at 4°C and 25°C.

As displayed in Figure 3, the size of micelles in each group showed nanolevel around 200 nm at 4°C. Further to compare P3L7-1105, P3L7-1405, and P3L7-1705, the size of micelles increases along with the increase in hydrophobic chain. In addition, the size of micelles in P0L10 showed greater size than the other groups. The aggregation of micelle in each group showed increase obviously when temperature was at 25°C. These results suggested that the aggregation of micelles appeared when temperature increased.

### 3.2. Characterization of the Thermosensitive Hydrogel

**Figure 4:**
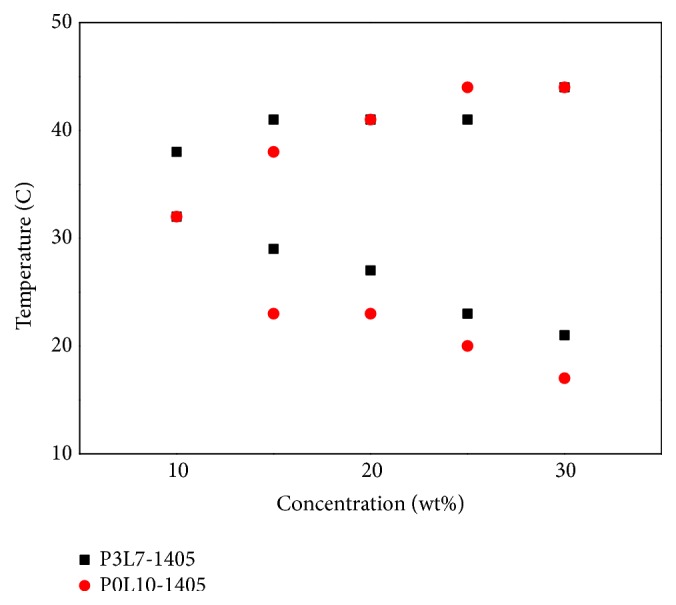
Sol-gel transition profile of copolymer solutions at P0L10-1405 and P3L7-1405.

**Figure 5:**
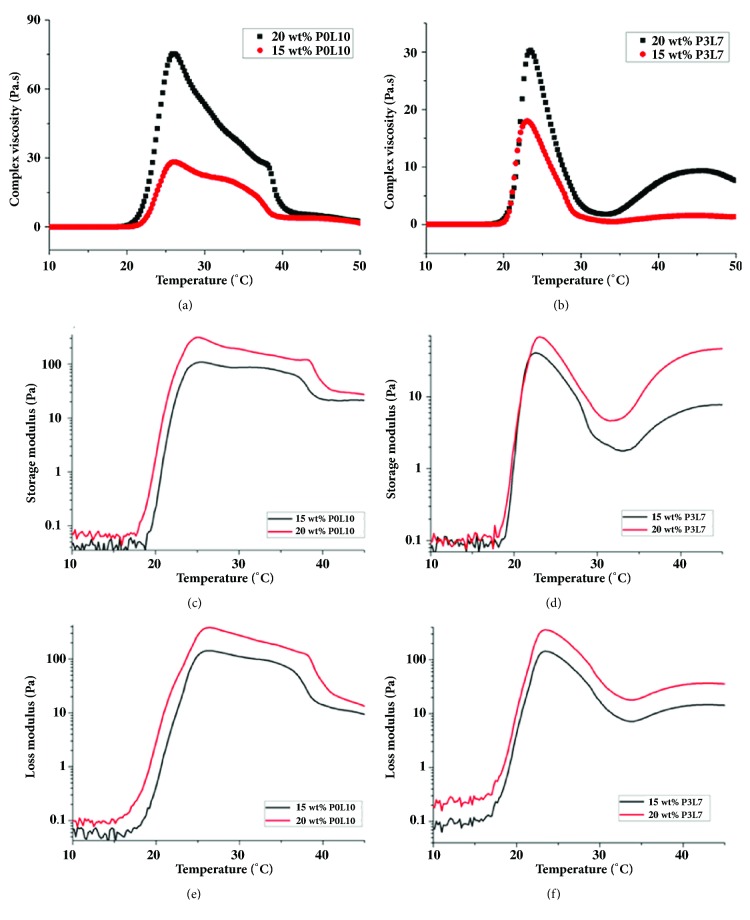
The viscosity (a and b) and mechanical strength (c–f) of 20 wt% mPDLA copolymer hydrogels. a, c, and e for P0L10; b, d, and f for P3L7.

Sol-gel transition tests were performed with copolymer solutions of 15 and 20 wt%. Among the diblock copolymers (Table 1), P3L7-1105 and P3L7-1705 could not form the gel structure possibly due to the weak hydrophobicity in the polymer chain. P3L7-1405 showed good solubility in water after mixing and was a translucent emulsion solution at 4°C. The phase transition diagrams of P0L10-1405 and P3L7-1405 are shown in Figure 4. The transition temperature of sol-gel process is a critical factor for application to the human body. The range of critical gelation concentration of P0L10 and P3L7-1405 was 10 to 15%. From 15 to 50°C, hydrogels exhibited three physical states: solution, gel, and precipitate (Figure 4). According to the experimental results in Figure 5, P3L7-1405 showed a great sol-gel-sol status. Therefore, this copolymer (P3L7-1405, named P3L7) was further tested in all experiments below. The mechanical characteristics of the P0L10 and P3L7 copolymers that are affected by the temperature were measured using a rheometer.

As displayed in Figures 5(a) and 5(b), the viscosities of copolymers at low temperature were around 1 Pa.s. With the increase of temperature to 19°C, the viscosities of copolymers started to increase. The maximal viscosity of copolymer (around 75 Pa.s) achieved when the temperature was increased to 23°C. The results in Figures 5(c)–5(f) and Table 2 show that the temperature for G' (storage modulus) was higher at 37°C than that for G” (loss modulus, tan *δ* < 1), and the temperature for G” was higher than that for G' at 20°C (tan *δ* > 1). When the temperature increased to 37°C, the gel became more stable. These results meant that the copolymer was able to perform a sol-gel transition within 20°C to 37°C.

### 3.3. Swelling Ratio

**Figure 6:**
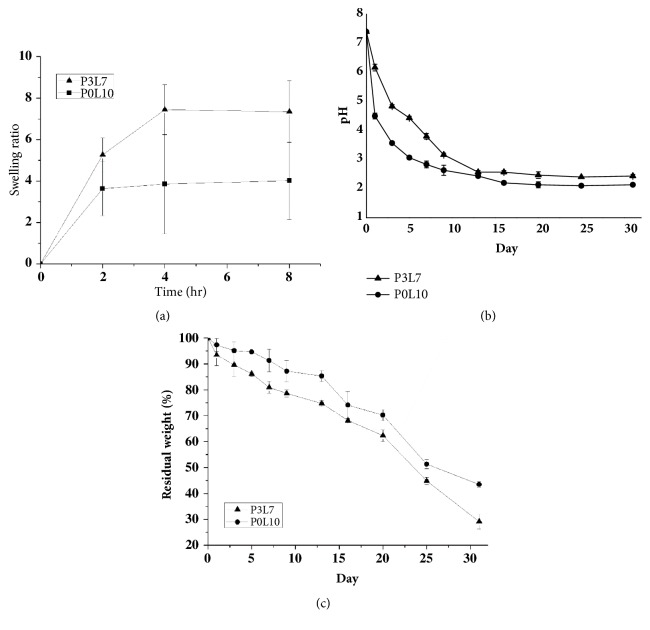
Swelling ratio of 20 wt% mPDLA copolymer hydrogels dispersed in DMEM (a). Degradation profile of 20% hydrogels in PBS: pH of surrounding medium (b) and percent residual weight (c). Triplicates were used for each experiment and values were expressed as mean ± SD.

The swelling ratio of the P3L7-1405 hydrogel, which has a larger hydrophilic block, was 2-fold higher than that of the P0L10 hydrogels (Figure 6(a)). A large hydrogel volume was observed for the P3L7-1405 hydrogel to hold a large amount of water when compared to that seen for the P0L10 hydrogel. Therefore, it is suggested that P3L7-1405 would provide an environment that is preferred by cells.

### 3.4. *In Vitro *Degradation of the Hydrogel

The degradation process of the hydrogel at 20 wt% mPDLAs was primarily a result of the hydrolysis mechanism (Figure 6(b)). According to Figure 5(a), the pH value of P3L7 was substantially lower than that of P0L10. However, the degradation process for P3L7 slowed down after 1 week, and no significant difference for pH value was found until day 31 when compared P3L7 (pH 2.6) and P0L10 (pH 2.4). The residual weight of 20 wt% mPDLAs was measured for 30 days. As seen in Figure 6(c), the experimental results showed that the degradation process for P3L7 was a little faster than P0L10. Until day 31, the residual weights of P0L10 and P3L7 were around 45% and 30%, respectively.

### 3.5. Viability of Encapsulated Chondrocytes

**Figure 7:**
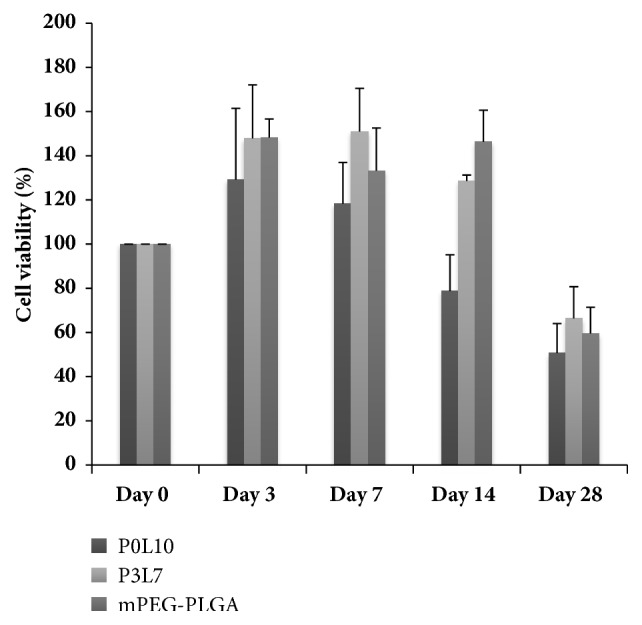
Percentage of cell viability of encapsulated chondrocytes in 20% hydrogels. Triplicates were used for each experiment and values were expressed as mean ± SD. Values of P3L7 group were higher than mPEG-PLGA and P0L10 (p < 0.05). Values of mPEG-PLGA were higher than and P0L10 (p < 0.05).

Cell viability was analyzed using the MTT assay (Figure 7). 20% copolymer hydrogels prepared in DMEM were homogenously mixed with chondrocytes and allowed to gel at 37°C. According to the experimental results in Figure 7, both P3L7 and mPEG-PLGA groups showed cell proliferation during 2 weeks (*p* < 0.05). However, the P0L10 hydrogel showed the contradictory result that the cell proliferation declined substantially from the first week to the end of culture (*p* < 0.05). This result suggested that P0L10 was not suitable for chondrocyte growth. Therefore, the following experiments were based on P3L7 and mPEG-PLGA hydrogels. Chondrocytes were distributed homogenously in the hydrogel pores either after 2 weeks or 4 weeks. Besides, the morphology of chondrocytes that were cultivated in the hydrogel showed a round shape that was different from those cultivated in the monolayer flask. This result indicated that chondrocytes encapsulated in the P3L7 hydrogel could maintain their chondrogenic properties.

**Figure 8:**
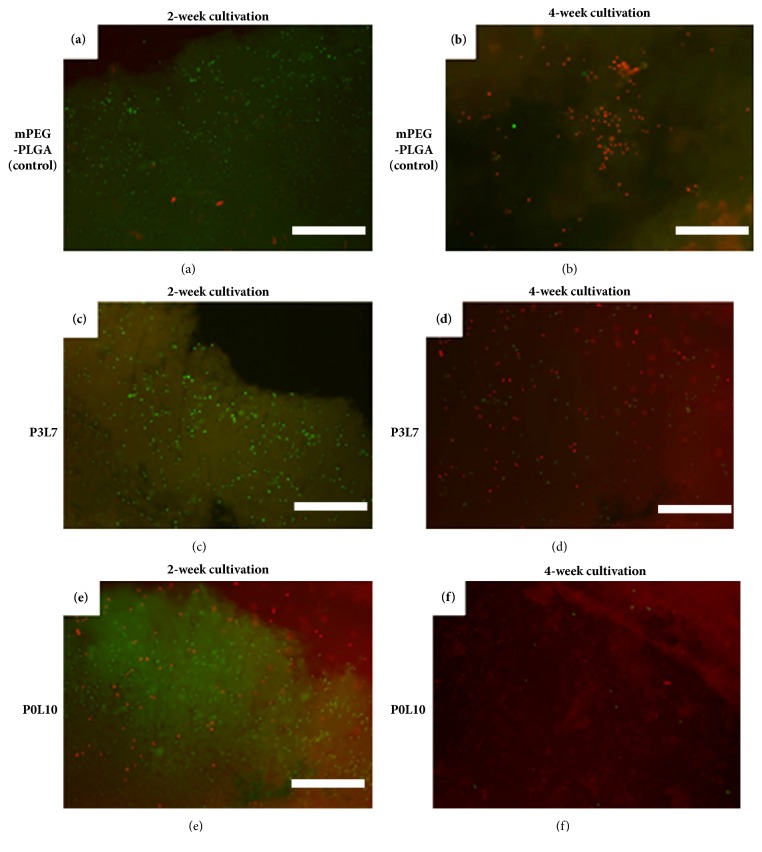
LIVE/DEAD stain of encapsulated chondrocytes in 20% hydrogels for 2 weeks (a, c, and e) and 4 weeks (b, d, and f). a and b for mPEG-PLGA; c and d for P3L7; e and f for P0L10. Scale bars = 200 *μ*m.

LIVE/DEAD staining of the encapsulated cells in mPEG-PLGA, P3L7 and P0L10 groups, respectively, were carried out for cell viability after 2-week and 4-week cultivations. After a 2-week cultivation period, the staining results showed more live cells than apoptotic cells in mPEG-PLGA and P3L7 groups (Figures 8(a) and 8(c)). However, P0L10 showed opposite results (Figure 8(e)). Four weeks later, the number of apoptotic cells in each group increased significantly. Among them, P0L10 showed the less live cells when to compare mPEG-PLGA and P3L7 groups (Figures 8(b), 8(d), and 8(f)). These staining results are consistent with the MTT data. These results also indicate that a hydrophilic environment is more suitable for chondrocytes.

### 3.6. Biochemical Analysis

**Figure 9:**
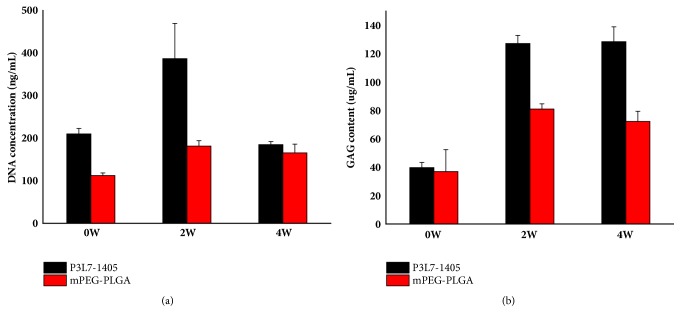
Biochemical analysis of cell-laden 20% hydrogels: (a) DNA content; (b) total GAGs content (per construct). Triplicates were used for each experiment and values were expressed as mean ± SD. Values of initial cell proliferation and GAG secretion in P3L7 were higher than in mPEG-PLGA groups.

Total DNA in each cell-laden hydrogel was quantified using the Hoechst 33258 stain and related to the cell number by dividing the DNA content with 7.7 pg per chondrocyte. The DNA data were taken as a measure of proliferation. According to the results in Figure 9(a), chondrocytes in P3L7 and mPEG-PLGA exhibited cell proliferation from the beginning up to week 2. Initial cell proliferation in the P3L7 group was higher than that in the mPEG-PLGA group (*p* < 0.05). However, the cell proliferation in the P3L7 group showed a substantial decrease from week 2 to the end of the experiment. Aside from cell proliferation, biochemical analysis was also performed to determine the capacity of cells to secrete ECM components (GAGs) when the chondrocytes were cultivated in the hydrogel. As depicted in Figure 9(b), both groups showed a capacity for GAG secretion, which was higher in the P3L7 group (128 *μ*g/mL) than that in the mPEG-PLGA group (85 *μ*g/mL) in the first 2 weeks (*p* < 0.05). However, no obvious increase was observed in the two groups for the rest of the cultivation period.

## 4. Discussion

The low self-healing capacity of cartilage regeneration in the current therapies is limited by several factors, including avascularity, easy formation of fibrocartilage, limited number of chondrocytes, and the defect size of cartilage [12–14]. Over the past decades, injectable thermosensitive hydrogels have attracted great attention as superior carriers for injectable drug delivery systems that are potentially useful in minimally invasive surgery [15]. Besides, thermosensitive hydrogels have shown potential to cartilage repair because of their ability to gel in situ and maintain cells within the defect. Hydrogels can be composed of natural polymeric, including chitosan and collagen, and synthetic polymers such as PEG-based hydrogels [16–18]. In this study, mPDLA has been synthesized, characterized, and evaluated for cell viability, cell proliferation, and chondrogenesis of chondrocytes.

Polyester-amide systems have become a promising biodegradable synthetic polymer with valuable properties by the combination of both polyesters (i.e., biodegradability) and polyamides (i.e. high thermal stability and high tensile strength) [19]. The most common polyamide to synthesize polyester-amides is nylon materials. Among them, 2-pyrrolidone is synthesized by ring-opening polymerization. Reports showed 2-pyrrolidone can be biodegraded in the natural environment as well as in organisms [20, 21]. The presence of the *α*-amino acid contributes to better polymer-cell interactions and allows the introduction of functional groups thus enhancing the overall biodegradability of the material [22].

Reports showed four different morphological forms (i.e., poly(l-lactic acid)(PLLA), poly(d-lactic acid) (PDLA), the racemic poly(d,l-lactic acid) (PDLLA) and meso-PLA) can be synthesized either from the polycondensation of lactic acid (LA) or from the ring-opening polymerization of lactide [23]. Only PLLA and PDLLA have been extensively applied for load bearing applications and drug delivery purposes in the literature [19]. Our study provided an alternative choice (i.e. PDLA) for polyester-amide system in the biomedical field.

To obtain the optimal ratio for mPDLA polyester-amide system using a sol-gel process, copolymers of several molecular weights were prepared based on their hydrophilic and hydrophobic balance. The rationale for sol-gel phase transition is that a higher concentration of diblock copolymer leads to more micelles in the solution, to further easily result in the aggregation of micelles when the transition temperature increases [6]. In general, the range of gelling temperature for P3L7-1405 and P0L10 was 15 to 50°C, and the range of the lowest gelling concentration was from 10 to 15% (Figure 4). To compare phase transition diagrams of P0L10 and P3L7-1405, the gelling temperature of P3L7-1405 was higher than that of P0L10. This difference can be attributed to the effect of hydrogen bonding. The diblock copolymer dissolves in water by hydrogen bonding. The functional group of NH_2_ from the PD monomer in water leads to an increased water affinity due to hydrogen bonding. Therefore, hydrophobic forces lead to the aggregation of micelles into a gel under relatively high temperature [24]. Furthermore, the aggregation of micelles into a gel resulted in an increase in viscosity when so-gel status occurred (Figures 5(a) and 5(b)). Moreover, because the hydrophobic force of P0L10 was stronger than that of P3L7, the mechanical strength of P3L7 was lower than that of P0L10 (Figures 5(c)–5(f)). Furthermore, based on the experimental results obtained using a rheometer, mPDLA showed thermoresponsive properties with the viscosity and mechanical strength varying with the temperature.

The level of swelling ratio for a hydrogel might be affected by the chemical structure and the intermolecular orientation of a polymer, and the porosity of hydrogel [25]. The particle size of micelle in the P3L7 polymer was notably greater than P0L10 (Figure 2). Accordingly, the results suggest that the high level of swelling ratio in the P3L7 polymer was due to the loose intermolecular orientation of the P3L7 polymer (Figure 6(a)).

Degradation behavior plays an important role for applying hydrogels to cell proliferation, ECM secretion, and tissue regeneration. The mPDLA degradation initially occurred by hydrolysis of the ester linkage of mPEG, followed by degradation at the hydrophobic segments. The results for in vitro degradation of hydrogel (Figure 6(c)) suggest that the faster degradation process for P3L7 than for P0L10 is due to the functional group of NH_2_ from the PD monomer that formed a hydrogen bond with water molecules. Thermosensitive hydrogels are made of polymer chains that possess both moderate hydrophilic and hydrophobic groups. Hence, polymer chains with too many hydrophilic segments would result in faster degradation due to the interaction between water molecules and hydrophilic segments [26]. Additionally, PD monomer decreased the LA crystallization level. Our results are consistent with the report showing that the LA degradation process was associated with the decrease of its crystallization level.

To apply a hydrogel in vivo, cell encapsulation and initial pH of the copolymer solution should be close to the physiological condition for cell viability and nutrient/waste transportation [9]. Additionally, cell growth, differentiation, and ECM secretion of mesenchymal stem cells are facilitated by a fine-tuned microenvironment. The results for the MTT analysis (Figure 7) showed that P3L7 induced higher cell viability than either P0L10 or mPEG-PLGA during the culture period (*p* < 0.05). These results are also consistent with the degradation behavior (as shown in Figure 6(c)) suggesting that byproducts from P0L10 hydrolysis might lead to a pH value that is lower than that of P3L7 and mPEG-PLGA; thus, byproducts affect the viability of chondrocytes. Moreover, Gan et al. showed that HepG2 cell growth was inhibited by a high degree of gelation [27]. In other words, a higher swelling ratio of hydrogel suggests a better potential for cell encapsulation in terms of nutrient/waste transportation. The results shown in Figure 6(a) coincided with those of the LIVE/DEAD staining (as shown in Figure 8). P3L7 with a higher level of swelling ratio presented with more live chondrocytes when compared to P0L10 and mPEG-PLGA. In addition, LIVE/DEAD staining confirmed the cell homogeneity in hydrogel, as well as the maintenance of the round morphology. These results also indicate that a hydrophilic environment is more suitable for chondrocytes.

As depicted in Figure 9(a), the trend in cell proliferation of both groups resembled that of the MTT analysis (Figure 7). Appropriate cell-to-cell contact for signal transportation also plays a role in adequate proliferation. The cell proliferation in P3L7 increased during the first two weeks and then showed a marked decrease by week 4, which may be due to the escape of cells through the larger pore size, because of the higher swelling ratio. The other reason for declined proliferation might be attributed to the accumulation of ECM, which hinders nutrient transport to the cells and causes further cell death (i.e., the necrosis of cells inside the hydrogel) and elimination from the hydrogel over time. In addition, the reason might result in ECM content maintained from week 2 to week 4. GAG is one major component in ECM of cartilage tissue and is evaluated as a marker for chondrogenesis. Our results showed that chondrocytes that were cultivated in the P3L7 hydrogel showed higher GAG secretion when compared to in mPEG-PLGA, which may be due to the high swelling ratio. Though no direct evidence (i.e., by scanning electron microscope) proved the pore size in the P3L7 hydrogel was higher than mPEG-PLGA hydrogel. Indirect evidence might provide a hint for this study that a higher swelling ratio led to more accessible pore volume, as well as larger interconnected pores and space for GAG secretion [9]. The larger pore size in the P3L7 hydrogel may provide more spaces for mature chondrocytes to secrete enough GAGs than in mPEG-PLGA hydrogel. Our results are consistent with this report.

## 5. Conclusions

A series of diblock poly(ester-amide) copolymers, mPDLA, was synthesized by ring-opening polymerization of mPEG550, D,L-lactide (LA) and 2-pyrrolidone (PD). The copolymers were characterized by 1H-NMR, FTIR spectroscopy, and GPC. The ratio of monomers in mPDLA was [PD]/[LA] = 30/70, and the target molecular weight of the hydrophobic segments was 1105, 1405, and 1705. Diblock copolymers formed nanomicelles at low concentrations in the aqueous phase. As the temperature increased, micelle aggregation was observed by DLS. P3L7-1405 solution underwent a sol-to-gel phase transition, which was confirmed by the test tube inverting method. Viscosity and mechanical properties of the copolymer solution varied with temperature, indicative of the formation of a gel. As an injectable scaffold, the viability, cell proliferation, and chondrogenesis of chondrocytes encapsulated in the mPDLA hydrogel were investigated. MTT and DNA quantification showed proliferation of cells within 2 weeks. By LIVE/DEAD staining, we could confirm that the mPDLA hydrogel presented a great chondrogenic response. Moreover, ECM content was significantly increased within the first 2 weeks. From above results, we deduced that this thermosensitive hydrogel could be suitable as an injectable scaffold for cartilage tissue engineering.

## Figures and Tables

**Table 1:** The reaction ratio and molecular weights calculated by ^1^H-NMR and GPC.

Sample	PD/LA (theoretical)	PD/LA (practical)	Mn^a^	Mn^b^	Mw^b^	PDI^b^
P0L10	0/100	0/100	2206	1523	1923	1.26
P3L7-1105	30/70	21/79	1518	1340	1460	1.09
P3L7-1405	30/70	19/81	2262	1631	1807	1.11
P3L7-1705	30/70	15/85	2539	1747	1994	1.14

^a^ Determined by ^1^H-NMR.

^b^ Determined by GPC.

**Table 2:** The difference of G' (storage modulus) and G” (loss modulus) in between 20°C and 37°C.

	20°C			37°C		
	G' (Pa)	G” (Pa)	tan *δ*	G' (Pa)	G” (Pa)	tan *δ*
P0L10	0.2333	0.4787	2.0518	27.7909	17.9542	0.6460
P3L7	0.3382	0.4808	1.4216	12.4076	5.1665	0.4164

## Data Availability

The data used to support the findings of this study are available from the corresponding author upon request.
